# Challenges in developing methods for quantifying the effects of weather and climate on water-associated diseases: A systematic review

**DOI:** 10.1371/journal.pntd.0005659

**Published:** 2017-06-12

**Authors:** Giovanni Lo Iacono, Ben Armstrong, Lora E. Fleming, Richard Elson, Sari Kovats, Sotiris Vardoulakis, Gordon L. Nichols

**Affiliations:** 1 Chemical and Environmental Effects Department, Centre for Radiation, Chemical and Environmental Hazards, Public Health England, Chilton, United Kingdom; 2 Department of Social and Environmental Health Research, London School of Hygiene and Tropical Medicine, London, United Kingdom; 3 European Centre for Environment and Human Health, University of Exeter Medical School, Truro, Cornwall, United Kingdom; 4 Gastrointestinal Infections, National Infection Service, Public Health England, London, United Kingdom; 5 Institute of Occupational Medicine, Edinburgh, United Kingdom; 6 University of East Anglia, Norwich, United Kingdom; 7 University of Thessaly, Larissa, Thessaly, Greece; University of California Berkeley, UNITED STATES

## Abstract

Infectious diseases attributable to unsafe water supply, sanitation and hygiene (*e*.*g*. Cholera, Leptospirosis, Giardiasis) remain an important cause of morbidity and mortality, especially in low-income countries. Climate and weather factors are known to affect the transmission and distribution of infectious diseases and statistical and mathematical modelling are continuously developing to investigate the impact of weather and climate on water-associated diseases. There have been little critical analyses of the methodological approaches. Our objective is to review and summarize statistical and modelling methods used to investigate the effects of weather and climate on infectious diseases associated with water, in order to identify limitations and knowledge gaps in developing of new methods. We conducted a systematic review of English-language papers published from 2000 to 2015. Search terms included concepts related to water-associated diseases, weather and climate, statistical, epidemiological and modelling methods. We found 102 full text papers that met our criteria and were included in the analysis. The most commonly used methods were grouped in two clusters: *process-based models* (PBM) and *time series and spatial epidemiology* (TS-SE). In general, PBM methods were employed when the bio-physical mechanism of the pathogen under study was relatively well known (*e*.*g*. *Vibrio cholerae*); TS-SE tended to be used when the specific environmental mechanisms were unclear (*e*.*g*. *Campylobacter*). Important data and methodological challenges emerged, with implications for surveillance and control of water-associated infections. The most common limitations comprised: non-inclusion of key factors (*e*.*g*. biological mechanism, demographic heterogeneity, human behavior), reporting bias, poor data quality, and collinearity in exposures. Furthermore, the methods often did not distinguish among the multiple sources of time-lags (*e*.*g*. patient physiology, reporting bias, healthcare access) between environmental drivers/exposures and disease detection. Key areas of future research include: disentangling the complex effects of weather/climate on each exposure-health outcome pathway (*e*.*g*. person-to-person vs environment-to-person), and linking weather data to individual cases longitudinally.

## Author summary

Unsafe water supplies, limited sanitation and poor hygiene are still important causes of infectious disease (*e*.*g*. Cholera, Leptospirosis, Giardiasis), especially in low-income countries. Climate and weather affect the transmission and distribution of infectious diseases. Therefore, scientists are continuously developing new analysis methods to investigate the impacts of weather and climate on infectious disease, and particularly, on those associated with water. As these methods are based on an imperfect representation of the real world, they are inevitably subjected to many challenges. Based on a systematic review of the literature, we identified seven important challenges for scientists who develop new analysis methods.

## Introduction

The seasonal and geographic distributions of infectious diseases are currently among the best indications of an association with weather and climate. The literature on climate effects is expanding in response to concerns about global climate change. The significance of the methods and data available is not only confined to the technical procedural aspects; methods and data also impact on the formulation of the specific scientific questions, their selection, and the development of hypotheses. Although our understanding of how weather and climate affect diseases has improved, the wide range of research methods applied make it difficult to get a robust overview of the state of research.

**Fig 1:**
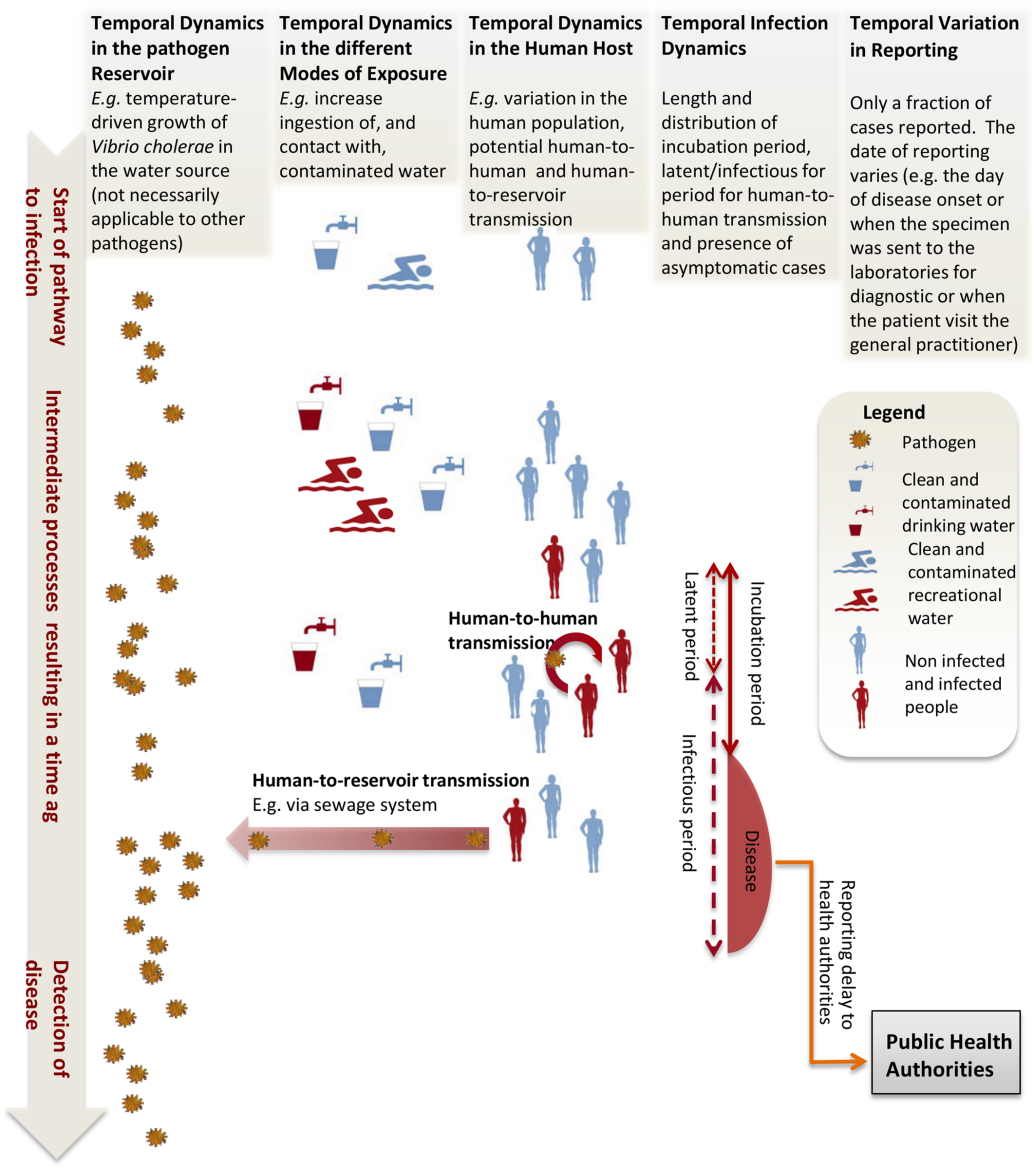
Illustration of the steps and potential pathways from being exposed to the pathogen reservoir to detection of disease. The red (blue) taps and swimmers represent contaminated (uncontaminated) drinking and recreational water. The red (blue) silhouette represents infected (not-infected) humans. Here and throughout, any kind of environment-containing pathogens that can serve as a medium for transmission (*e*.*g*. drinking water, sewage system) is referred to as “pathogen reservoir”; any form of direct or indirect contact with such medium, irrespective of the presence of the pathogen, is referred to as an “exposure”. According to this conceptual scheme, a disease-free situation is the combination of negligible pathogen population in, and/or negligible exposure of susceptible individuals to, the pathogen reservoir. Infections arise from increased interactions of exposed susceptibles with the pathogen reservoir. This can be caused by a growth in the pathogen population (driven, for example, by temperature) and/or larger exposure to the pathogen. An increase in the exposure can be directly or indirectly driven by meteorological/climate variables (*e*.*g*. high temperature increasing the risk of drinking contaminated water), environmental causes (*e*.*g*. poor water drainage management due to land use), and behavioural and/or socio-economic factors (*e*.*g*. recreational activity in unclean water). Changes in the population of susceptibles (for example due to immigration, loss of immunity and/or human-to-human transmission) can alter the patterns of exposure.

The relationship between climate/weather and infectious diseases is complex (*e*.*g*.[1]), as shown in the example illustrated in Fig 1. Investigating the effects of weather and climate on infectious diseases requires the ability to: i) disentangle concurrent modes of transmissions (*e*.*g*. environmental from human-to-human transmission); ii) tease apart the individual effects of multiple exposures at different temporal and spatial scales; iii) identify and separate socio-economic drivers and behavioural causes; iv) integrate all these different processes into a unified perspective; v) attribute changes in disease to observed environmental changes (such as climate change); and vi) quantify infectious disease burden resulting from current social, economic and environmental conditions which can help to project the future disease burden resulting from these changes. These are difficult methodological and conceptual demands, and the scientific and public health community could benefit from a critical overview of the available research methods and the challenges ahead.

In this paper, we focus on the particularly important, especially in developing countries, class of infectious diseases associated with water (including those classified as neglected tropical diseases (NTD) according to the World Health Organisation (WHO) [2] the US Centers for Disease Control and Prevention (CDC) [3]) and the journal Plos NTD [4]) (Table 1). According to WHO estimates, 1.1 billion people globally drink water that is of at least ‘moderate’ risk of faecal contamination [5], and 842,000 annual deaths are attributable to unsafe water supply, sanitation and hygiene (including 361,000 deaths of children under age five), mostly in lower income countries [6,7].

**Table 1:** Organisms causing diseases related to water (excluding vector borne diseases). The symbol ● specifies the known routes of transmission (not exclusively); (●) specifies the probable route of transmission but no direct evidence available. The last column indicates which organism is classified as Neglected Tropical disease, according to the World Health Organisation (W) [2], the Centers for Disease Control and Prevention (C) [3], and the journal PLOS Neglected Tropical Diseases (P) [4]. The different routes of transmission are: A) Drinking water borne; B) Water washed (reduced water access); C) Water based; D) Foodborne through water; E) Water infecting wounds; F) Bathing water transmission; G) Respiratory waterborne; H) Toxic poisoning through a bloom; I) Infection or disease related to damp; J) Medical water or solutions.

Organism	Routes of Transmission										Organism type	Details of disease & organism & water relationship	Classified as Neglected Tropical Disease
	A)	B)	C)	D)	E)	F)	G)	H)	I)	J)			
	Drinking water-borne	Water washed (reduced water access)	Water based	Food-borne through water	Water infecting wounds	Bathing water	Respirat. water-borne	Toxic poisoning through a bloom	Infection or disease related to damp	Medical water or solutions			
***Acanthamoeba polyphaga***	●		●			●					Protozoan	Primary amoebic meningoencephalitis, eye infections	
**Adenovirus**	●					●					Virus	Diarrhoea; respiratory infection	
**Alexandrium spp.**			●	●				●			Dinoflagellate	Shellfish poisoning from bioaccumulation	
***Alternaria* spp.**									●		Fungus	Alimentary toxic aleukia associated with mouldy grain crops	
***Anabena* spp.**			●					●			Cyanobacteria	Exposure to contaminated dialysis, drinking and bathing water	
***Ancylostoma* spp.**	(●)										Roundworm	Hookworm infection	WP
***Angiostrongylus spp*.**			●	●							Roundworm	Larvae develop in aquatic animals	WCP
***Anisakis spp*.**			●	●							Roundworm	Lifecycle in fish	W
***Aphanizomenenon* spp.**			●					●			Cyanobacteria	Exposure to contaminated dialysis, drinking and bathing water	
***Arcobacter* spp.**	(●)										Bacteria	Contamination of drinking water	
***Ascaris lumbricoides***	●										Roundworm	Drinking water contaminated by nightsoil	WCP
***Aspergillus spp*.**				●							Fungus	Linked to Balkan endemic nephropathy from mouldy grain	
**Astrovirus**	●										Virus	Contamination of drinking water	
***Baylisascaris procyonis***	●										Roundworm	Infection from racoons through water?	WP
***Burkholderia pseudomallei***	●		●				●				Bacteria	Parasite contamination through untreated drinking water	
***Burkholderia cepacia***	●										Bacteria	Possible water transmission	
***Balamuthia mandrillaris***	●		●								Protozoa	Parasite contamination through untreated soil/water	P
***Balantidium coli***	(●)										Protozoa	Parasite contamination through untreated drinking water	
***Blastocystis hominis***	●										Protozoa	Parasite contamination through untreated drinking water	
***Bunostomum phlebotomum***	(●)										Roundworm	Larva migrans from food or water consumption	WP
***Campylobacter* spp.**						●					Bacteria	Contamination of unchlorinated drinking water	
***Capillaria philippinensis***			●	●							Roundworm	Transmission through fish, crabs and snails	
***Chilomastix mesnili***			●								Protozoa	Parasite contamination through untreated drinking water	
***Chlamydia trachomatis***		●									Bacteria	Transmission through flies as a result of lack of water to wash	WCP
***Chryseobacterium***										●	Bacteria	Contaminated water for medical uses	
***Cladosporium spp*.**									●		Fungus	Implicated in alimentary toxic aleukia.	
***Clostridium botulinum***				●							Bacteria	Botulism in injecting drug users; Washwater contaminating cans	
**Coronavirus (SARS)**							(●)				Virus	Aerosolisation of water	
***Cryptosporidium* spp.**	●					●					Protozoa	Contamination of drinking and bathing water	
***Cyclospora cayetanensis***	●										Protozoa	Contamination of water used for irrigation/spraying/washing	
***Cylindrospermopsis raciborskii***								●			Cyanobacteria	Exposure to contaminated dialysis, drinking and bathing water	
***Dientamoeba fragilis***	●										Protozoa	Parasite contamination through untreated drinking water	
***Dinophysis* spp.**			●								Dinoflagellate	Diarrhoretic shellfish poisoning	
***Diphyllobothrium latum***			●								Flatworm	Infection through uncooked fish	WP
***Dracunculus medinensis***	●		●								Flatworm	Through consumption of drinking water containing copepods	WCP
***Echinococcus* spp.**	●										Flatworm	Transmission through water consumption	WCP
***Echinostoma spp*.**			●	●							Flatworm	Infection through consumption of uncooked molluscs or amphibians	WP
***Encephalitozoon spp*.**	●										Fungus	Parasite contamination through untreated drinking water	
***Entamoeba histolytica***	●										Protozoa	Diarrhoea, dysentery & abscess	P
***Enterobius vermicularis***	(●)										Roundworm	Parasite contamination through untreated drinking water	
***Enterocytozoon bieneusi***	●										Fungus	Parasite contamination through untreated drinking water	
**Enteroviruses including ECHO coxsackie, polio etc.**	●										Virus	Sewage contamination of drinking water	
**E. coli O157 & others**	●										Bacteria	Contamination of drinking water	P
** Enteropathogenic (EPEC)**	●										Bacteria	Contamination of drinking water	P
** enterotoxigenic (ETEC)**	●										Bacteria	Contamination of drinking water	P
** Enteroaggregative (EAggEC)**	●										Bacteria	Contamination of drinking water	P
** enteroinvasive (EIEC)**	●										Bacteria	Contamination of drinking water	P
***Fasciola gigantica***			●	●							Flatworm	Infection through contaminated aquatic plants	WCP
***Fasciola heppatica***			●	●							Flatworm	Infection through contaminated aquatic plants	WCP
***Fasciolopsis buski***			●	●							Flatworm	Infection through contaminated aquatic plants	WCP
***Fusarium* spp.**									●		Fungus	implicated in alimentary toxic aleukia.	
***Gambierdiscus* spp.**			●	●							Dinoflagellate	Ciguatera fish poisoning from bioaccumulation	
***Giardia* sp.**						●					Protozoa	Contamination of drinking and recreational water	P
***Gnathostoma spp***			●								Roundworm	Transmission through eating uncooked fish or frogs	W
***Gongylonema pulchrum***	●										Roundworm	Rare infection linked to water consumption	WP
***Gonyaulax tamarensis***			●	●							Dinoflagellate	Ciguatera fish poisoning from bioaccumulation	
***Gymnodinium spp*.**			●	●							Dinoflagellate	Shellfish poisoning from bioaccumulation	
***Helicobacter pylori***	●										Bacteria	Transmission by water in developing countries	
**Hepatitis A**	●										Virus	Contamination of unchlorinated drinking water	
**Hepatitis E**	●										Virus	Contamination of unchlorinated drinking water	
**Heterophyes heterophyes**			●	●							Flatworm	Infect ion from uncooked fish	WP
***Isospora belli***	●										Protozoa	Parasite contamination through untreated drinking water	
***Karenia spp*.**			●	●							Dinoflagellate	Shellfish poisoning from bioaccumulation	
***Legionella pneumophila***			●			●					Bacteria	Contamination of warm water systems in buildings	
***Leptospira* spp.**	●				●	●					Bacteria	Water contamination from wild and agricultural animals	P
***Lyngbya majuscule***			●					●			Cyanobacteria	Contact exposure to algae	
***Microcystis aeruginosa***			●					●			Cyanobacteria	Exposure to contaminated dialysis, drinking and bathing water	
***Mycobacterium avium***										●	Bacteria	Contamination of water systems	
***M*. *avium P*aratuberculosis**	(●)										Bacteria	Natural water and food contaminated by cow and sheep faeces	
***Mycobacterium chelonae***	●		●							●	Bacteria	Contamination of water systems	
***Mycobacterium fortuitum***	●		●							●	Bacteria	Contamination of water systems	
***Mycobacterium marinum***	●		●								Bacteria	Contaminated fishtanks	
***Mycobacterium ulcerans***			●		●						Bacteria	Wound infections with contamination from water plants	WCP
***Naegleria fowleri***			●								Protozoa	Contamination of natural thermal waters	
***Nanophyetus salmincola***			●	●							Flatworm	Infection from uncooked fish	W
***Necator americanus***											Roundworm	Infection from soil or water	WCP
***Nitzschia navis-varingica***			●	●							Diatom	Shellfish poisoning from bioaccumulation	
***Nodularia spumigena***			●					●			Cyanobacteria	Exposure to contaminated dialysis, drinking and bathing water	
**Norovirus**	●										Virus	Contamination of unchlorinated drinking water	
***Opisthorchis* spp.**			●								Flatworm	Infection from uncooked fish	WP
***Oscillatoria* spp.**			●					●			Cyanobacteria	Exposure to contaminated dialysis, drinking and bathing water	
***Ostreopsis* spp.**			●				●	●			Dinoflagellate	Respiratory exposure to algal blooms	
***Paragonimous spp*.**			●	●							Flatworm	Infection from uncooked crabs	WP
***Penicillium spp*.**									●		Fungus	Toxins implicated in Balkan endemic nephropathy and alimentary toxic aleukia.	
***Phormidium favosum***			●					●			Cyanobacteria	Exposure to contaminated dialysis, drinking and bathing water	
***Plesiomonas shigelloides***	●										Bacteria	Contamination of natural waters	
***Procentrum spp*.**			●	●							Dinoflagellate	Shellfish poisoning from bioaccumulation	
***Protoperidinium crassipes***			●	●							Dinoflagellate	Shellfish poisoning from bioaccumulation	
***Pseudomonas aeruginosa***										●	Bacteria	Ear, eye and skin infections from bathing and other waters	
***Pseudo-nitzschia spp*.**			●	●							Diatom	Shellfish poisoning from bioaccumulation	
***Pseudoterranova spp*.**			●	●							Roundworm	Infection through consuming raw fish	WP
***Pyrodinium spp*.**			●	●							Dinoflagellate	Shellfish poisoning from bioaccumulation	
***Rhinosporidium seeberi***			●				●				Protozoa	Infection linked to bathing and washing	
**Rotavirus**	●										Virus	Contamination of drinking water	
***Stenotrophomonas maltophilia***											Bacteria	Contaminated hospital water systems	
***Salmonella* spp.**											Bacteria	Contamination of untreated drinking water	P
***Salmonella* Typhi *&* Paratyphi**	●										Bacteria	Contamination of untreated drinking water	P
***Schistosoma intercalatum***			●			●					Flatworm	Infection through the skin from working or bathing in water	WCP
***Schistosoma haematobium***			●			●					Flatworm	Infection through the skin from working/bathing in water	WCP
***Schistosoma mansoni***			●			●					Flatworm	Infection through the skin from working/bathing in water	WCP
***Schistosoma mekongi***			●			●					Flatworm	Infection through the skin from working/bathing in water	WCP
***Schistosoma spindale***			●			●					Flatworm	Infection through the skin from working/bathing in water	WCP
**Sapovirus**	●										Virus	Contamination of untreated drinking water	
***Sarcocystis hominis***											Protozoa	Contamination of natural waters with dog faeces	
***Shigella* spp.**	●	●									Bacteria	Contamination of untreated drinking and bathing water	P
***Spirillum minus***	●										Bacteria	Potable water contaminated by rodents	
***Spirometra spp*.**	●		●								Flatworm	Sparganosis infection from copepods in drinking water	WCP
***Taenia solium***	●										Flatworm	Ova of *T*. *solium* can cause cysticercosis through ingestion in drinking water	WCP
***Trichodesmium erythraeum***			●	●							Cyanobacteria	Exposure to contaminated dialysis, drinking and bathing water	
***Trichuris trichiura***	●										Protozoa	Can be transmitted through drinking water	WCP
***Toxocara canis***	(●)										Roundworm	Possible transmission from contaminated soil and water	P
***Toxoplasma gondii***	●										Protozoa	Infection through oocyst contamination of drinking water	
***Trichobilharzia regenti***			●			●					Flatworm	Infection through the skin from working/bathing in water	WP
***Uncinaria* spp.**	(●)										Roundworm	Larva migrans. Oral transmission through water likely	W
***Vibrio choleraee***	●		●	●							Bacteria	Transmission from estuarine, human and food sources	P
***Vibrio parahaemolyticus***			●	●							Bacteria	Contamination of shellfish	
***Vibrio vulnificus***			●	●	●						Bacteria	Contamination of shellfish	
***Vibrio* spp. (non-*V*. *cholerae*)**	●		●	●							Bacteria	Contamination of shellfish	
***Yersinia enterocolitica***	●										Bacteria	Contamination of untreated water by rodents	
***Yersinia pseudotuberculosis***	●										Bacteria	Contamination of untreated water by rodents	

Infectious diseases associated with water are classified as follows: “water-system-related” infections (i.e. *via* aerosols from poorly managed cooling systems, *e*.*g*. Legionellosis), “water-based” infections (i.e. *via* aquatic vectors or intermediate in hosts, *e*.*g*. Schistosomiasis), “water-borne” infections (i.e. *via* bacterial, parasitic and viral oral-faecal infection through ingestion, *e*.*g*. cholera), and “water-washed” infections (i.e. infections arising from poor hygiene due to insufficient water, these can also include oral-faecal infection, *e*.*g*. hookworm) [8] (see Table 1). Here and throughout, we use the expression “water-associated” to refer to these latter classes of diseases. Of note, we excluded diseases arising from ingestion/contact with inorganic and other chemical compounds (*e*.*g*. arsenic) and vector-borne infections linked with water (*e*.*g*. malaria, rift valley fever, river blindness) from the “water-associated” diseases.

This Review is not a prescriptive guideline of available methods for a range of problems. We reviewed and summarised the methods used to investigate the effects of weather and climate on infectious diseases associated with water, with the objective of identifying the challenges that scientists are facing when develop new analysis methods. We focused on quantitative analytical approaches, such as: mathematical models, statistical analysis, computational techniques, numerical simulations, epidemiological models and computer-generated agent based models. We excluded purely descriptive observational studies [9].

Discriminating between studies which build explanatory models versus create predictive models is particularly important in statistical modelling [10]. We however avoided this way of grouping. The dichotomy explanatory *vs* predictive models might be clear from an epistemological point of view [10], nevertheless, we have found it really challenging to rigorously separate papers according to this classification. For most papers, a formal distinction is often impossible as the causal relationships are inferred/discussed from the patterns captured from predictive models, and vice versa the hypothetical-deductive models (*e*.*g*. driven by causal relationships), could both be able to predict a range of future scenarios.

## Methods

### Search strategy, selection criteria and methods

The methods for the systematic review followed the Guidelines developed by the Cochrane Collaboration [11]. We searched for English language articles published from 2000 to April 2015. The following databases were searched: Scopus, Medline, EMBASE, CINAHL, Cochrane Library, Global Health and LILACS bibliographic databases. The literature after April 2015 was also monitored using a daily email alert tool provided by Google Scholar (searching for “water borne disease” and “water related disease”) to identify potential papers adopting newly-developed methods not covered by the initial search.

We used search terms related to water-associated diseases (*e*.*g*. “water transmission” OR “contaminated fresh water” OR “unsafe water supply” etc.) and quantitative methodologies (*e*.*g*. “mathematical epidemiology” OR “simulation”) and weather and climate. The full list of search terms is in the Supporting Information, S2 Text. Papers were reviewed by two people (GL and GN). As the pool of returned papers was quite large, we decided to not use additional specific search terms for pathogens (*e*.*g*. `cholera’, `rotavirus’) or diagnosis/symptoms (*e*.*g*. 'diarrhoea', 'gastroenteritis'), as this would require a subjective list of potential pathogens and introduce unnecessary bias in the selection of the papers. We included articles that: i) were published in peer reviewed journals; ii) included an infectious disease in human beings; and iii) developed new methods and/or applied established methods to investigate the effects of weather and/or climate on infectious diseases (including papers for which weather and climate variables were among other equally important factors driving disease transmission).

The final set of papers was archived in EndNote (see Supporting Information, S1 Table). We identified specific questions related to the nature of the methods, their range, applicability and limitations (Table 2) that we wanted the Review to address. We then created a spreadsheet consisting of records (rows) corresponding to each paper in our final database, and columns to address the specific questions. Analysis was done in R open source analytic software [12].

**Table 2:** List of scientific questions and related notes.

	Questions	Notes
**1)**	What are the main water-related pathogens investigated and where do they occur?	Estimate the number of studies for each disease/pathogens under investigation. Ascertain the countries where the disease occurred.
**2)**	What methods have been used?	Ascertain the key epidemiological methods developed and used so far. Classify the methods in terms of general approaches: such as descriptive phenomenology, process based models (*e*.*g*. mechanistic compartmental models or agent based models), statistical analysis of empirical data (*e*.*g*. time series analysis) etc.
**3)**	Is the method applied to investigate the effect of climate change or weather or both?	Assess if the method is actually or potentially applied to climate change and/or weather
**4)**	Does the type of method depend on the disease/pathogen under investigation?	Ascertain if there is a preferential use of the methods towards particular disease/pathogen and identify possible explanations
**5)**	What are other key features of the methods?	Identify the specific environmental factors that the method is focusing on (*e*.*g*. temperature and rainfall). Ascertain if the method take into account: Temporal variations (*e*.*g*. seasonalities and El Niño weather cycles) Evolution of pathogen Human behaviour and/or social and political scenarios (*e*.*g*. national economic factors, conflicts, food production, human mobility, demography etc.) Any kind of heterogeneity (*e*.*g*. spatial variation, age group, household income) An explicit model for the pathogen dynamic in the reservoir
**6)**	How were the results assessed?	Establish if and how the method has been validated
**7)**	What are the limitations of the method according to the authors?	Describe the limitations of the methods identified by the authors

Papers were clustered according to the methodology used. More precisely, for each paper we identified the list of technical keywords associated with the methods, including both general concepts (*e*.*g*. “time series analysis”) and sub-analysis terms (*e*.*g*. “partial autocorrelation function”); the full list of technical keywords is presented in the Supporting Information, S1 Table. Papers that share the same keyword are often connected. Consequently, analytical methods that are likely to be used together in the same papers tend to cluster. Analysis was done by using the “igraph” package in R [13].

## Results

**Fig 2:**
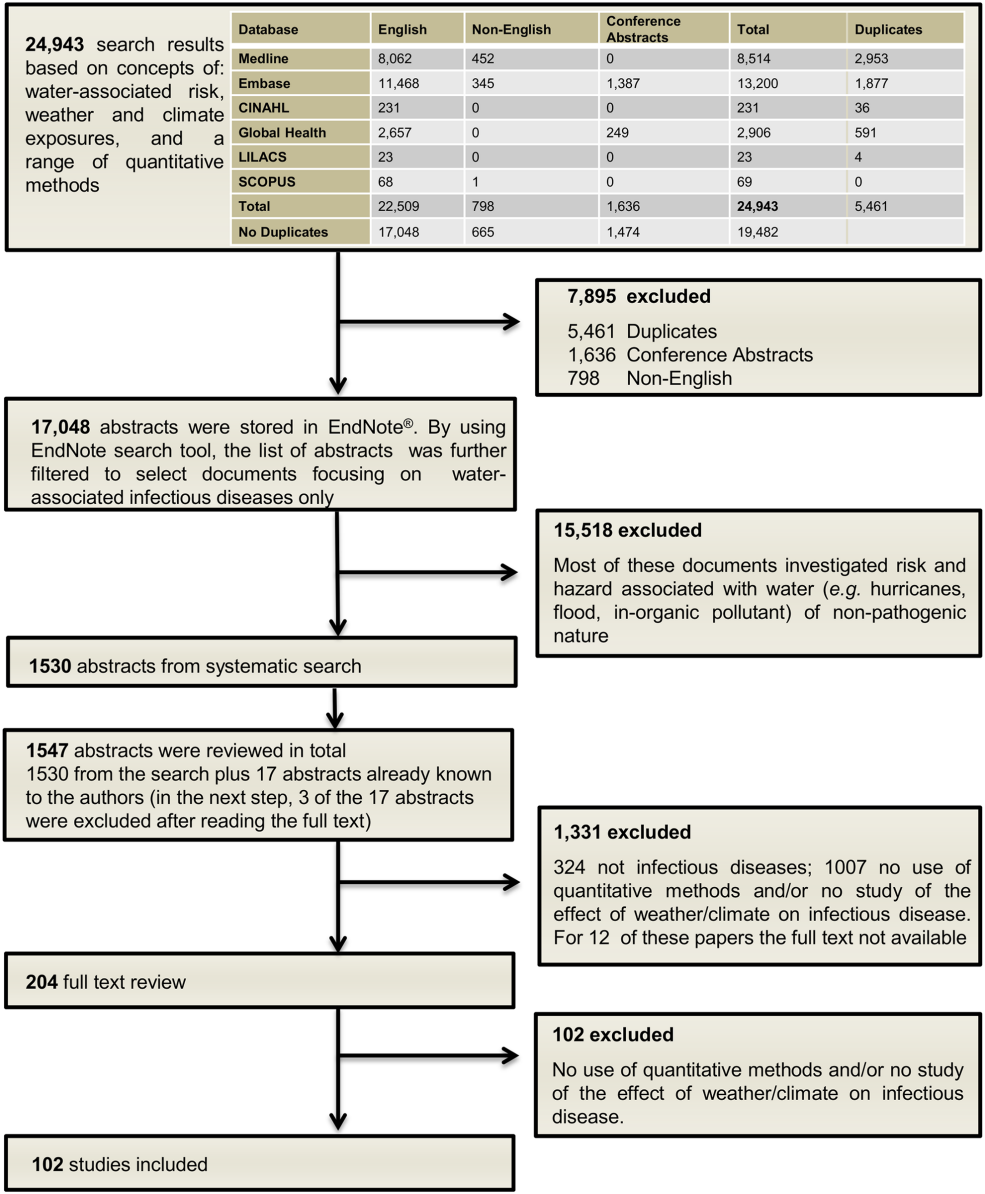
Flow chart describing the selection process of all abstracts.

Overall, 102 papers were included in the analysis (Fig 2). Analysis of the findings and synthesis of the challenges in formulating new methods are summarised below.

### Analysis: Seven particular scientific questions addressed by the review

#### 1) What are the main water-associated pathogens investigated and where do they occur?

**Fig 3:**
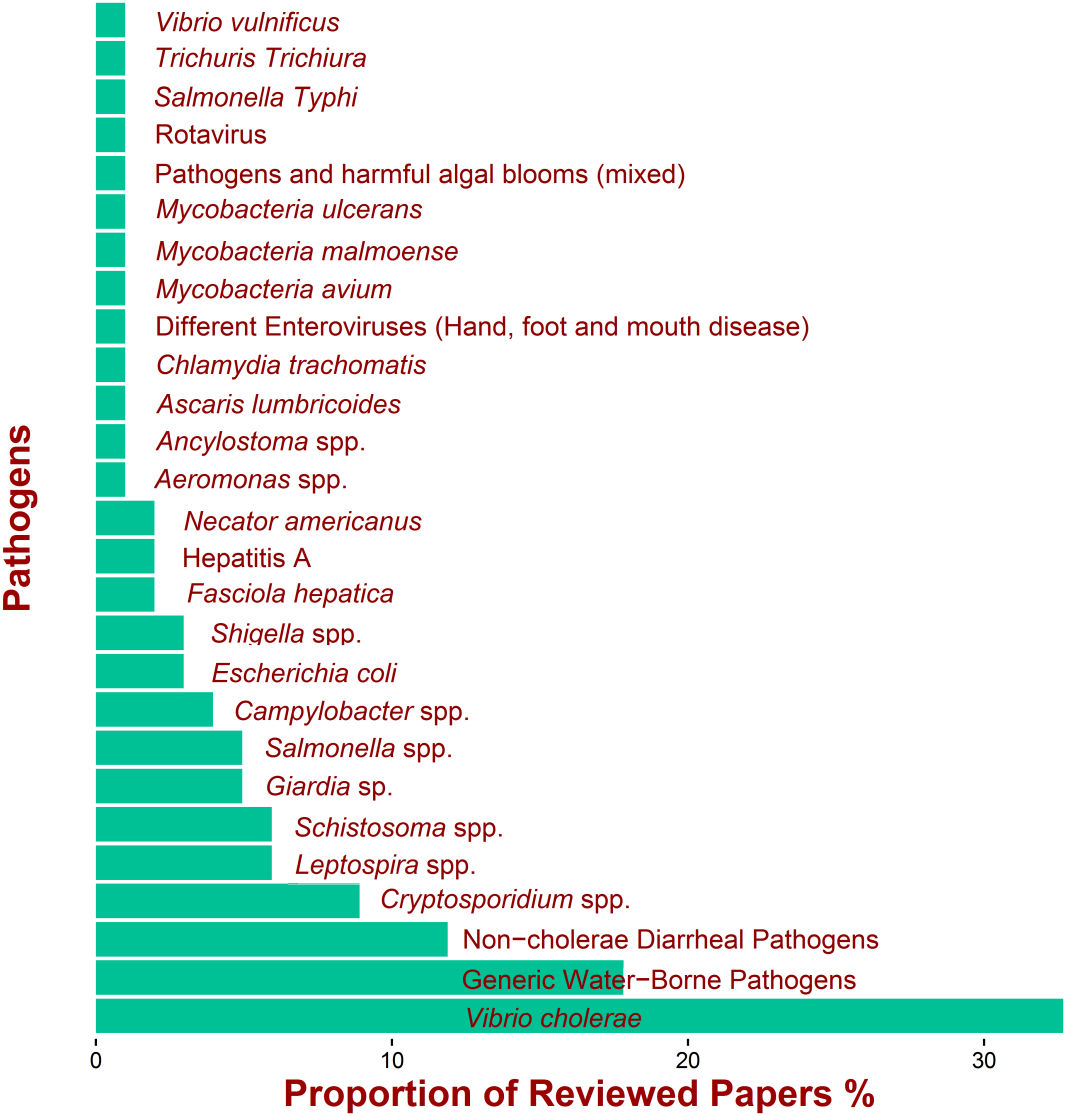
Proportion of papers investigating a particular pathogen or disease.

Fig 3 shows the frequency of pathogens in the final set of papers. *Vibrio cholerae* has been studied most for climate and weather effects. A significant proportion (approximately 20% of papers) focused on unspecified water-associate pathogens and these studies were mostly theoretical process-based models. The next significant categories of studies were papers that looked at diarrheal illness as a broad category based on health service data that did not include pathogen-specific information. In terms of pathogen -specific outcomes, the following pathogens were most studied (after *Vibrio cholerae*): C*ryptosporidium* spp., *Leptospira* spp., *Schistosoma* spp. *Giardia* sp. *and Salmonella* spp. Many of these are classified as NTD [2–4] *e*.*g*. *Vibrio cholerae*, *Leptospira* spp., *Schistosoma* spp. *Giardia* sp. (Table 1).

**Fig 4:**
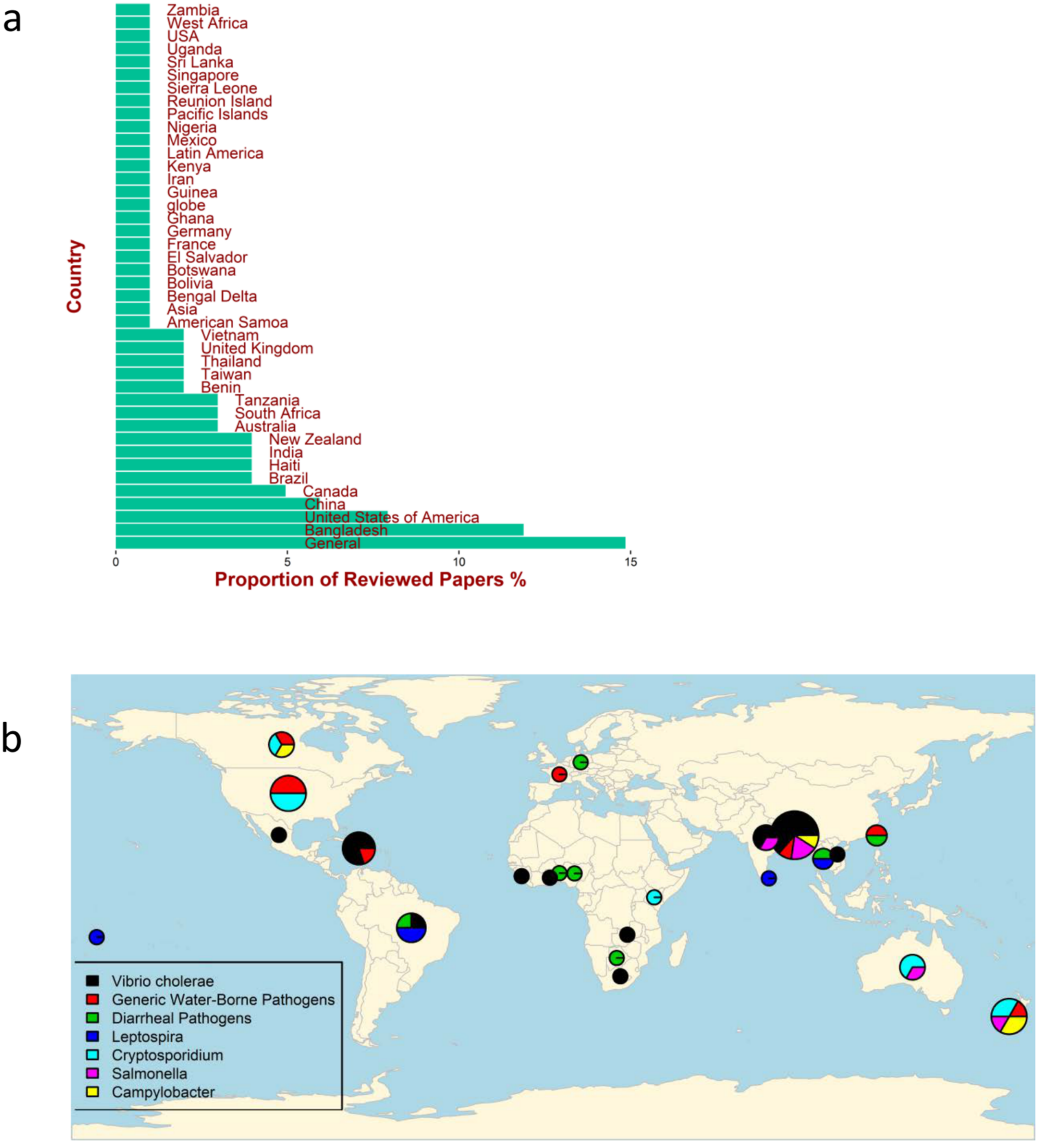
(a) Distribution of countries for which water-associated disease data were the focus of the reviewed papers (b) Geographic distribution of the 7 most commonly studied water-associated pathogens (resulting either in an epidemic or endemic situations) which were the focus of the reviewed papers. Each circle refers to specific countries, In particular, the largest circle in Asia, refers to Bangladesh.

Fig 4 shows the countries where the studies were based. A good proportion of these theoretical process-based models did not link their study with any data for diseases occurring in a particular country (therefore, the outcome was listed as “General” in Fig 4). The country with the most studies (about 10% of papers) was Bangladesh, mostly in relation to cholera, followed by studies on disease data collected in US, China and Canada (Fig 4).

Thus, the pathogens reported in these studies reflect geographic (origin of infections) and socio-economic (quality of data) features: studies on cholera were associated with low and middle income countries, while *Cryptosporidium* spp. and *Campylobacter* spp. were more likely reported in high income countries that have good laboratory-based passive surveillance systems (Fig 4).

#### 2) What methods have been used?

**Fig 5:**
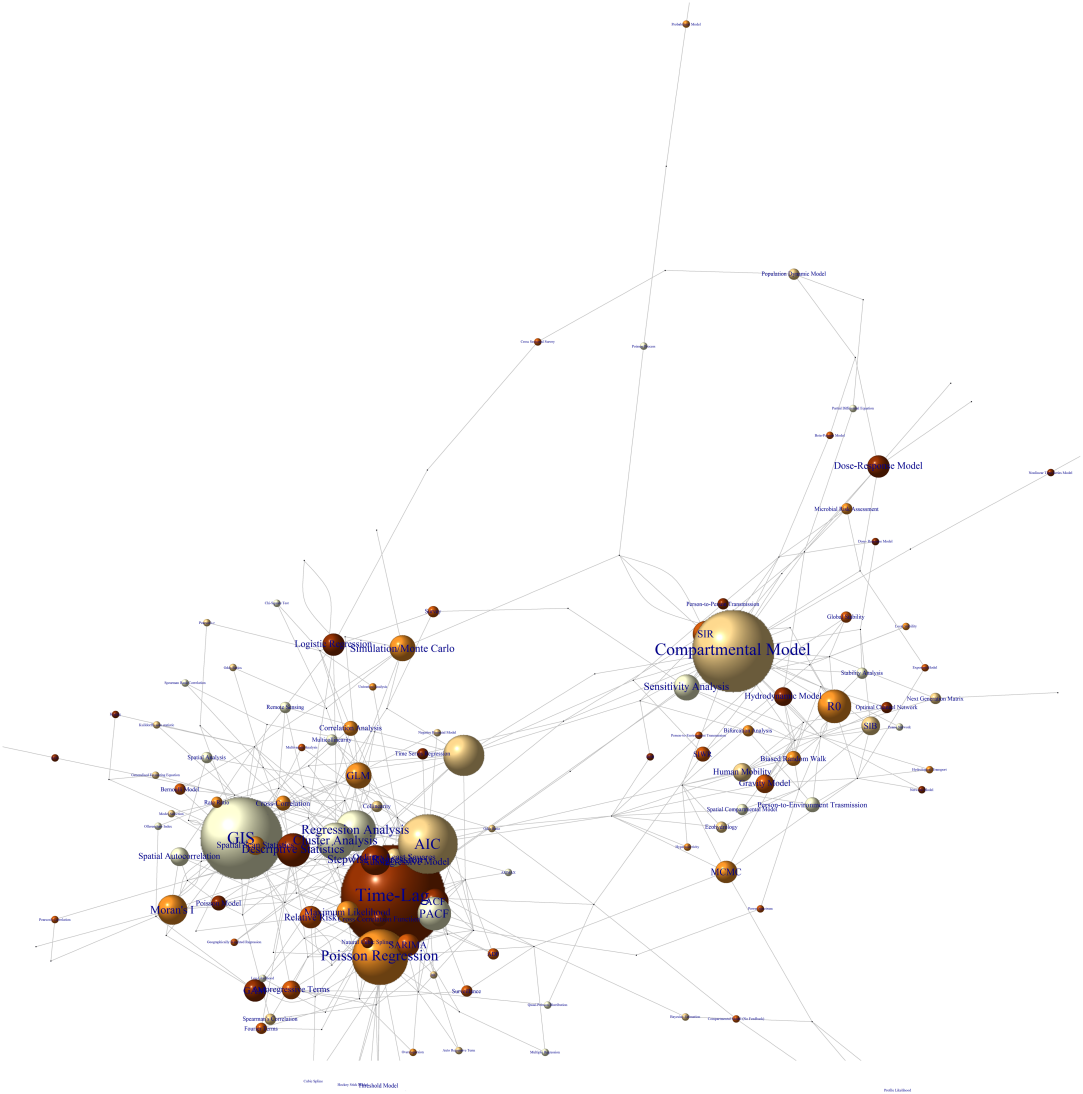
Cluster analysis of methods. Each dot corresponds to a reviewed paper; the brown bubbles correspond to the keywords describing the techniques. A connection between a paper and a keyword occurs when the related technique is used. The size of the bubble increases (logarithmically) with the number of papers citing the keyword. For visual purpose only, i) the bubbles are displayed with different shades of brown and ii) the technical keywords (listed in S1 Table in the Supporting Information) describing methods used by only one paper are not displayed (the full set is shown in S1 Fig in the Supporting Information). The graph was produced by using the i-graph package[13] in R.

The set of all technical keywords describing the methods used in at least two papers is displayed in a keyword network in Fig 5 (a high-resolution image for the methods used in each paper can be found in the Supporting Information, S1 Fig, see also S1 Table). The figure suggests that the most commonly used methods can be grouped into two main clusters:

- *Process-Based Models* (PBM), which are typically described by mechanistic compartmental models [14],
- *Time series and spatial epidemiology* (TS-SE) such as time series and regression analysis [15] and spatial methods typically based on geographic information system (GIS) [16].

These clusters can overlap. For examples, instances of spatial compartmental models have been developed.

**Fig 6:**
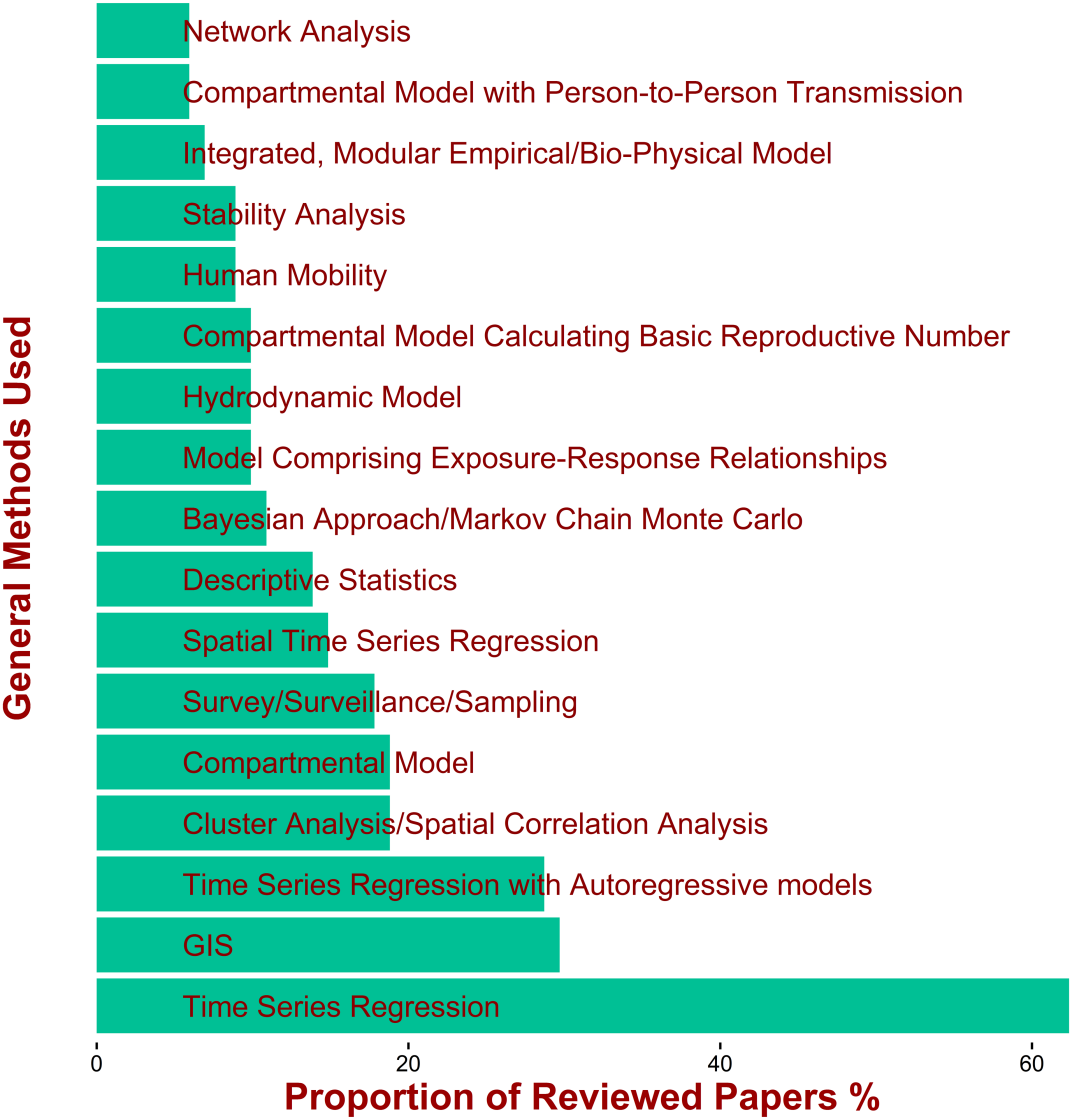
The most common, general methods used in the reviewed papers (listed in S1 Table in the supporting information).

Fig 5 is perhaps the most objective way of representing the methods used in the reviewed papers as it is based simply on the technical keywords recorded by their authors. As the technical keywords can be very specific, the next exercise was to identify the general methods used in the papers. A list of the most common general methods is shown in Fig 6 and in S1 Table in the Supporting Information. The entries in Fig 6 and S1 Table do not reflect an established”taxonomy” of the methods (which is not available in the literature); they are mainly guided by the patterns which emerged from keyword network (Fig 5) and selected based on their potential relevance for the study of the effects of weather and climate. For example, a close inspection of Fig 5 suggests that a substantial number of papers in the PBM clusters employed “Dose-Response Model” (which often use environmental variables, such as temperature as inputs); we therefore identified “Models comprising Exposure-Response Relantionship” as a general method.

The same principle guided the structure of Table 3, which presents some key features of the clusters of methods. In Table 3, however, we did not discuss methods such as “Descriptive Statistics” and “Survey/Surveillance/Sampling” since these were too generic in terms of their use for investigating the effect of weather and/or climate change on water-associated diseases. Conversely, the table contains additional entries that did not emerge from the patterns from Fig 5, but we recognized that they are important for such use (e.g. “Investigation of seasonality” and separation of “Time Series Regression” in short and long term studies).

**Table 3:** Discussion of some key features of the general methods and their relevance to the investigation of the effect of weather and/or climate change.

**PBM**	**Key aspects of the cluster potentially relevant to study the impact of weather/ climate change**	**Discussion**	**Use/Potential use of the method to investigate the effect of weather and/or climate change**
	Compartmental models, Spatial Compartmental Models (SIR, SIB, SIBR, etc.)	Compartmental models are based on a partition of the entire population into key epidemiological categories, for example Susceptible, Exposed, Infected, Recovered individuals [14]. When the models are applied to water-borne diseases, often include an additional compartment describing the dynamic of the pathogen population in the environment. This category is usually indicated by W (water) or B (bacteria). Infections occur when a susceptible individual comes in contact with this additional category of with the infected category (for human-to-human transmission). Infected people can excrete pathogens, and feed back into the environment compartment. The acronyms (SIR, SEIR, SIB etc.) reflect the specific epidemiological compartments considered. These models typically uses differential equations to estimate the number of individuals in each compartment at any time, but other techniques such as Agent Based Models or difference equations could be employed.	These models are fully specified by a set of parameters (*e*.*g*. transmission, recover, mortality rate etc.) that regulate the transition from one compartment to another. Many of these parameters depend on environmental variables such as temperature. When the functional form of the parameter *vs*. the environmental variable is known, investigation of the effect of weather and/or climate change on the epidemics is straightforward.
	Models Comprising Exposure-Response Relationship	The keywords describing approaches for exposure–response relationship (“Beta-Poisson models”, “Dose-Response models”, “Exposure Model”) also belong to the cluster PBM. In fact many papers that employ exposure–response relationship are integrated with compartmental models as a separate module governing the pathogen dynamics in the environment compartment.	In the present context, exposure-response relationships relate the magnitude of a stressor (*e*.*g*. concentration of a pathogen in the water reservoir) to the response of the receptor (*e*.*g*. probability of getting infected). The effect of weather/climate on the exposure-response curve can be direct (*e*.*g*. an increase in water load due to rainfall results in a diluted concentration affecting the response) or indirect (*e*.*g*. when the functional form of the exposure-response curve depends on weather/climatic variables)
	Stability Analysis	All papers employing stability analysis (identified by keywords “Global Stability”, “Local Stability”, “Bifurcation”) are connected within the cluster PBM. This is not surprising as standard CMs are based on sets of differential equations, which is the appropriate environment for stability analysis [85].	Stability analysis studies if all sufficiently small disturbances away from the equilibrium solution (*e*.*g*. a fixed or periodic population), damp out in time [86]. This property depends on the parameter of the system and a variation of a parameter of the system can lead to a transition from stability to instability (bifurcation). In the present context, a change in the environmental parameters (e.g. mean temperature, rainfall) might lead to a transition from disease free equilibrium to an endemic situation or to a variation in the periodicity.
	Compartmental Model Calculating Basic Reproductive Number	All papers calculating the reproductive numbers (“Basic Reproductive Number”, “Effective Reproductive Number” etc.) use PBM approaches. This is usually done analytically from the set of differential equations or by using the Next Generation Matrix approach [87].	The reproductive number, i.e. the number of secondary cases arising from a primary case under certain conditions, depends on the set of parameters. The approach can be used to investigate which range of the relevant environmental parameters might result in the reproductive number below or above one (i.e. the disease fades out or it establishes)
	Human Mobility	Human mobility is often neglected in epidemiological models, however, a small proportion of PBM-papers incorporate human mobility, for example, in the form of “gravity model” [88]. All the reviewed papers, however, focus only on short-term human mobility	Human mobility will be likely affected by changes in the environmental and socio-economic factors arising from climate change [89], with an impact on formation and severity of the spatio-temporal epidemic patterns.
	Hydrodynamics Model and Network Analysis	The detailed water flow of rivers, estuarine, pipe network etc. can be incorporated in PBM to assess spatio-temporal exposure to contaminated water. This is usually done by employing hydrogeological models or abstract network theory (see *e*.*g*. [90]) where the nodes of the graph represent communities and links represent their connection (hydrological but also due to human mobility).	Hydrodynamics models can take into account variations, due to *e*.*g*. rainfall, in the spatio-temporal exposure to the pathogen caused by variations in the contact with contaminated water or in the concentration of the pathogen dispersed in the water.
	Compartmental Model with person-to-person Transmission and also person-to-environment transmission	Person-to-person is an additional mode of transmission of many water-associated diseases, *e*.*g*. cholera. Many PBM address these processes by allowing infection transmission when a contact between a susceptible and infected person occur. Furthermore, the infected population can excrete pathogens back to the environment, for example via the sewage system. The process can be reproduced by letting the rate of growth of the pathogen population in the environment increases with the number of infected people.	The effects of climate and weather can have an indirect impact on these additional modes of transmission, for example by changing the population size (*e*.*g*. due to increased urbanization) and the rates of contact among people, and by altering the patterns of contact at people-environment interface (for instance, socio-economic factors impacting sewage treatment).
	Simulation/Agent Based Models	Only a few studies employed Agent Based Models. These are general methods mimicking the bio-physical processes with a computationally-aided set of autonomous, interacting agents. A key advantage is their ability to resolve heterogeneity in a population (which is not necessarily grouped into compartments).	Apart the potentially high computational costs, Agent Based Models are extremely flexible to incorporate specific effects of climate and weather for a variety of situations (e.g. changes in human behavior). These are particularly useful when the mathematical approach is less tractable.
**TS/SE**	**Key aspects of the cluster potentially relevant to study the impact of weather/ climate change**	**Discussion**	**Use/Potential use of the method to investigate the effect of weather and/or climate change**
	Investigation of seasonality	These studies seek to describe patterns of disease by season. Cosinor and spectral analyses may be used, but also simple tabulation of rates by season. However, in this review we did not find this particular type of studies.	Can give strong indirect information on dependence of disease on weather, but may be misleading if any other risk factors also vary seasonally (*e*.*g*. holidays).
	Time Series Regression with Auto-Regressive Models (Disease forecasting studies)	These studies, often using Box-Jenkins methods such as (S)ARIMA, and sometimes incorporating preceding weather as predictors, seek a method to obtain a forecast of disease in the short term future given the series up to the present.	Those forecasting studies that explicitly incorporate weather variables can indirectly provide information on weather-disease associations, but may not separate components that are causal from those due to co-varying factors (*e*.*g*. holidays)
	Time series regression I (studies of short term associations)	These studies seek to directly explore the dependence of disease patterns over time on preceding and concurrent weather. Their methods are based on standard GLMs (or occasionally GAMs), in particular Poisson and negative binomial models [15,91]. Most studies control bias from temporal trends and seasonal patterns not necessarily due to weather, and other measured time-varying risk factors. Some allow for residual autocorrelation either using (S)ARIMA or ad hoc approaches (see Time Series Regression with Auto-Regressive Models).	Dependence of disease on weather in the explicit focus of these studies. However, it is unclear how much they could be relied on to predict impact of climate change. One relevant limitation is that they largely ignore the complexity of the dependence as explored in the PBMs. For example very few allow for the variation in susceptible persons due to people becoming immune after contracting the disease.
	Time series regression II (studies of longer term associations)	Studies have explored in particular the association of medium-term weather cycles such as ENSO with disease. Methods options are as for other TSRs, though specifics not.	These have some advantages over TSR of short term associations in being closer to studying association of climate change on health, although they are subject to similar potential biases and their time scales are below the many-decade scale of anthropogenic climate change.
	Spatial Analysis (Cluster Analysis, Geographic information systems (GIS))	Most spatial methods cover three main areas: Descriptive or visual presentation of data (choropleth maps, GIS), tests for global or local clustering (Moran’s I, spatial scan statistics) and analysing point data to estimate the intensity of spatial processes (kriging, splines) [92–94].	GIS allows disease data to be linked to datasets (for example, meteorological data) on the basis of geographical location and time. These methods allow the examination of disease risk in relation to space, time and space-time and also allow the interpolation of data points from limited sampling data.
	Spatial Time Series Regression	Spatial time series regression (also called spatio-temporal regression) explores the dependence of infectious disease outcomes with weather when both are measured in units varying over both space and time.	Spatial time series regression maximises contrasts in exposure (variance) when data with both space and time are available. Its main challenges are to control for both spatially and temporally varying confounding variables and autocorrelation.
**Other**	Hybrid time series-PBM studies.	A few studies employed Hybrid time series-PBM studies. These are applications of “time series susceptible-infectious-recovered (immune)” (TSIR) PBMs incorporating dependence of force of infection on weather [24] overlap with TSR models [15].	We believe that these approaches have promise to inform dependence of disease on climate and climate change.
	Bayesian Approach/Markov Chain Montecarlo	An introduction on Markov Chain Monte Carlo (MCMC) methods, usually associated with Bayesian analysis, is presented in [95,96]. These methods are becoming increasingly popular as they allow estimations of parameters and associated uncertainties for large classes of complex models for which standard estimation is difficult if not impossible. Availability of open source software e.g. WinBugs [97] has largely promoted their use.	These methods are particularly suitable in the presence of complex mechanistic or statistical approaches with many unknown parameters (mirroring the complex impact of weather and/or climate change on water-associated diseases). An important limitation is the computational power required for some models.

*Process-based methods (PBM)*. Most PBM are based on compartmental models, i.e. a subdivision of the entire population into relevant epidemiological categories, such as susceptible, infected, and recovered people [14]. The population dynamics of each category is usually governed by a system of non-linear differential equations (with each single equation corresponding to the rate of change of each compartment). This class of models has been extended to stochastic, spatial and age-specific models. The compartmental models in our Review included an additional compartment describing the dynamic of the pathogen population in the environment (e.g. the concentration of the pathogen in the water reservoir) which is then linked in some way to temperature and/or rainfall factors (*e*.*g*., rainfall affects the volume of the water reservoir, which determines the dilution of the pathogen and, thus, the probability of contracting the disease; temperature affects the growth and survival of many free living pathogens, such as *E*. *coli*, in the water reservoir). An infection occurs when a susceptible person comes in contact with this additional category. Infected people can excrete pathogens, and feedback into the environment compartment (included in the majority of the PBM-papers), although for some infections there is no excretion and thus no feedback into the environment (e.g. *Legionella*). Ten percent of PBM-papers also included person-to-person transmission via contacts between susceptible and infected persons. The cluster PBM also contained sub-clusters: “Exposure–Response Relationship”, “Stability Analysis”, “Human Mobility”, and “Network Analysis” (Fig 5). Important features of the clusters are discussed in Table 3.

*Cluster TS-SE*. Time series regression (TSR) analysis [15,17] is one of the most common methods used by the papers reviewed to analyse temperature and rainfall exposures as they can vary over time. Many studies used generalized linear models (GLMs) and generalized additive models (GAMs) often included terms allowing for over-dispersion. Terms to control for seasonality (time stratified model, Fourier terms, spline functions) and autocorrelation terms are often included. In most cases, residual variation in the response variable (e.g. daily counts of disease occurrence) was modelled as a Poisson distribution, followed by negative binomial. The most common exposure factors were temperature and rainfall. Socio-economic indicators were often included in the analysis.

As noted in the systematic review of Imai and Hashizume [15], only a few studies included variations in the susceptible population over time, due to, for instance, changes in immunity following disease recovery. Autoregressive models (e.g. ARMA, ARIMA, SARIMA, ARIMAX), which intrinsically take into account correlation, were used in some studies (Fig 6), although many studies in the regression tradition used ad hoc approaches for this. These methods were often used to investigate the temporal lag between the exposure and the response variable (e.g. daily counts of disease occurrence).

Spatial methods for linking datasets use geographical information systems (GIS) to link disease data with information on socio-economic indicators, temperature and rainfall (or proxy indicators), and vegetation and land use data within the same geographical framework. Geo-referenced environmental data can be collected by remote sensing [16,18] and ground-station data.

Other less commonly used analytical methods included wavelet analysis [19], and the social science approach of participatory modelling [20].

#### 3) Is the method applied to investigate the effects of climate or weather?

Most of the reviewed papers (49% of the papers explicitly emphasized this application in the Abstract) investigated the effects of weather (i.e. short-term changes in the atmosphere, such as daily or weekly exposures to rainfall) on infectious disease. Only 9% of the reviewed papers applied the methodology to study the effects of climate, i.e. long-term averages of weather such as El Niño cycles (these applications are not mutually exclusive); and 7% used modelled future climate projections. Collinearity of exposures, i.e. highly correlated predictor variables in regression models, is an important limitation in weather and climate studies but was only explicitly identified as a limitation in 7% of studies).

#### 4) Does the type of method depend on the disease/pathogen under investigation?

Approximately 50% of papers investigating *Vibrio cholerae* employed methods based on compartmental models, followed by time-series/regression analysis and spatial/GIS analysis using cholera case observations. Similar patterns were observed for unspecified water-associated pathogens. As expected, for generic diarrheal pathogens (for which there is much more uncertainty about the causes), spatial/GIS and time-series/regression analysis were the most commonly used methods (but see discussion in [1] and [21]).

#### 5) Some key feature in the methods: e.g. What are the independent variables in the models? Does the model take into account seasonality?

**Fig 7:**
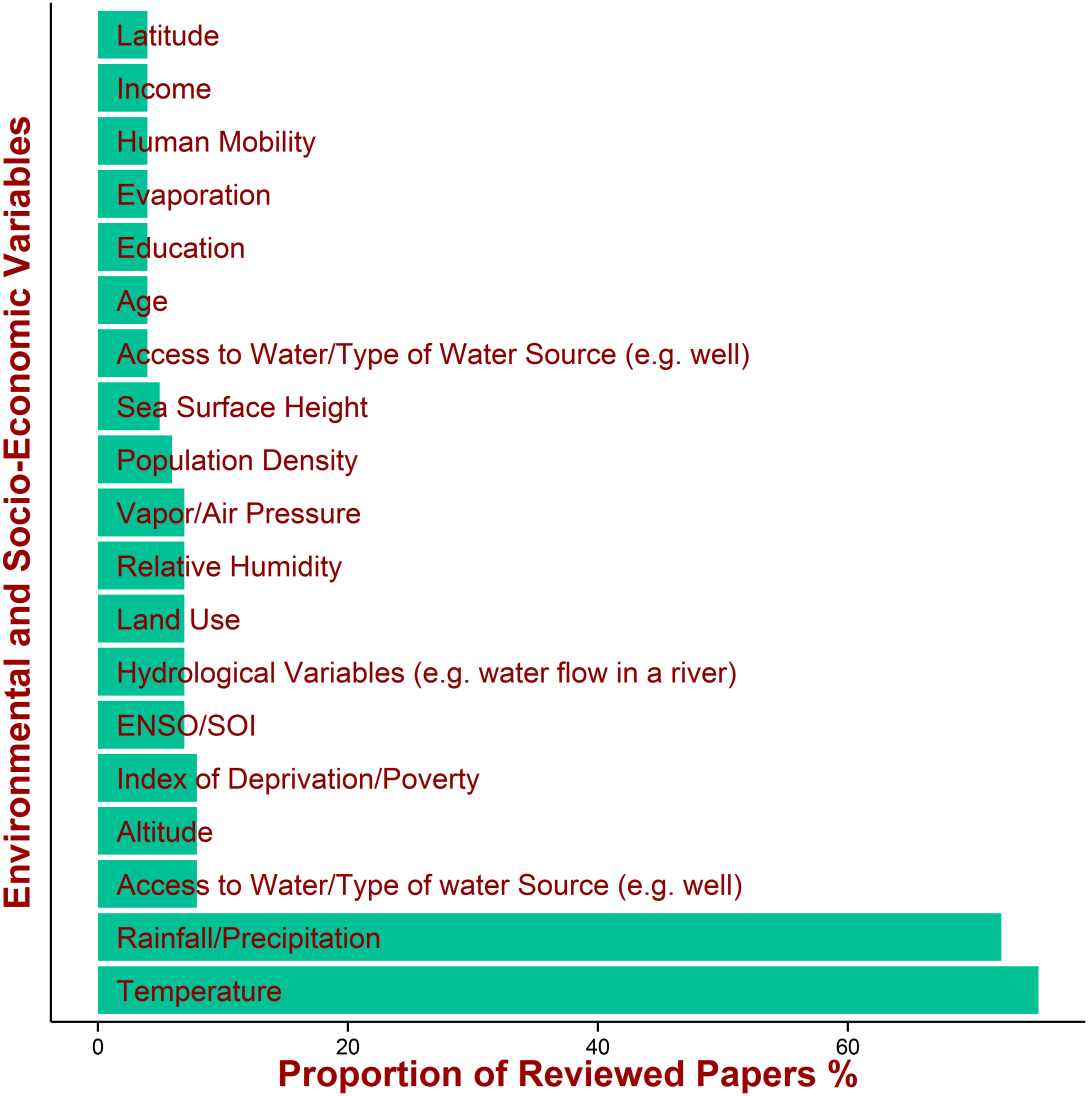
The most common environmental and socio-economic variables included in the reviewed papers.

More than 70% of studies included observed or modelled temperature and/or rainfall/precipitation data in their analysis. A smaller proportion of studies included (the inclusion is not mutually exclusive) other environmental factors (e.g., relative humidity, vapor air pressure, evaporation) and socio-economic indicators (e.g. access to water, index of poverty, age, education, human mobility) in the analysis (Fig 7).

Around 40% of the methods explicitly included the effects of seasonality (intra-annual climate variability). A small proportion of papers (7%) included the effects of El Niño/Southern Oscillation (ENSO) and North Atlantic Oscillations (NAO). Almost 40% of the methods took into account spatial variation. Only a small proportion of the methods explicitly modelled the pathogen dynamics in the wider environment or the specific water reservoir; a proportion of these studies (typically, theoretical works for a proof of concept) developed general methods without focusing on specific environmental variables, but the method could be potentially applied to investigate their effects.

#### 6) How were the results assessed?

The statistical methods used to fit a model with the observed data were assessed with information criteria (such as Akaike Information Criterion, AIC and Bayesian Information Criterion, BIC) in almost 20% of cases. In a significant proportion of papers (10%), the validation of the method was based on out-of-sample predictions, *i*.*e*. a subset of the data were used to train/calibrate the method (*e*.*g*. to estimate model parameters), and then the method was applied to the rest of the data. In some cases, there was no assessment of the methods. Situations where the methods did not require comparison with real data (e.g. theoretical works requiring solely logical demonstration of theorems) were also present.

#### 7) What are the method limitations for analysing climate and weather effects identified by the authors?

The lack of inclusion of relevant factors in the methods was the most common limitation acknowledged by the authors. These included: spatial and socio-economic heterogeneity, seasonality, changing immunity, and other environmental drivers. In almost 20% of papers, the authors identified reporting bias as a key limitation. Examples of reporting bias were: sample collections not properly designed (e.g. not stratified by age); voluntary internet-based survey reflecting survey respondents’ idiosyncrasies; and health-seeking behaviours and socio-economic factors affecting access to health facilities.

The poor quality of the data was another important source of limitation according to the authors, and this was explicitly mentioned in around 30% of reviewed papers. Typical examples of poor data quality were: low spatio-temporal resolution of the exposure data (e.g. environmental exposure covered a wide geographic area or linked to a single weather stations); lack of longitudinal data (only cross-sectional surveys were undertaken); and low accuracy of the data (e.g. reliance on proxy data, missing data due to asymptomatic or unobserved infections).

In 10% of cases, the methods were not able to explain the observed patterns in the disease outcome. The authors identified the absence of underlying mechanistic explanation as a problem in about 10% of the studies. In 10% of papers, the authors highlighted that the methods were calibrated only for a specific situation (e.g. a limited region), and the findings were not generalizable.

### Synthesis: Seven challenges in developing methods for quantifying the effects of weather and climate on water-associated diseases

#### 1) Disentangling multiple transmission pathways and identifying the bio-physical mechanism of how weather affects disease and seasonality

In general, the spread of many pathogens is subjected to concurrent modes of transmissions, as exemplified in Fig 1. For instance, cholera can be acquired from contamination of household water storage containers, food preparation, direct person-to-person contacts, and/or via contact with environmental reservoir with long pathogen persistence [22,23]. Identifying a potential signature for the particular pathways in the patterns of diseases is perhaps the ideal goal. In particular, to separate person-to-person transmission from other modes of disease transmissions a range of methods have been proposed, including nonlinear time series approaches linked with wavelet analysis [24] and mechanistic compartmental approaches [22,23]. Despite such efforts, isolating the contributions of person-to-person transmission on the burden of these diseases is a compelling problem not only for water-associated diseases but all infectious diseases [25,26].

Different transmission pathways can be strongly affected by weather and/or climate. For example, temperature may have direct effects on *Salmonella* bacterial proliferation at various stages in the food chain (including bacterial loads on raw food production, transport and inappropriate storage), and indirect effects on eating behaviours during hot days [27]. Rainfall might increase person-to-environment transmission of cholera, by facilitating the contamination of fresh water from the sewage system. Rainfall might also dilute the concentrations of the pathogens, reducing environment-to-person transmission [28]. Compartmental models have also been used to investigate the effects of pathogen dilution due to the seasonal variation of water volume (e.g. with monsoons) and the potential interactions with other environmental drivers (e.g. temperature [29,30] and the effects of human mobility) [31].

The key challenge ahead are increasing the awareness of the drivers of disease and a deeper integration with fields such as microbiology (e.g. identifying dose-response curves to use as input for modelling, potential coexistence of human-to-human transmission), social science (e.g. to identify and include in the methods social contacts, patterns of mobility, adaptation, etc.), and ecology (e.g. to understand and incorporate the dynamics of free living organisms in water).

#### 2) Reducing uncertainty in reporting

Measuring the ‘true’ incidence of disease, and therefore morbidity and mortality rates, is a common problem in epidemiology. This includes: the under-ascertainment arising when not all cases seek healthcare; under-reporting due to failure in the surveillance system; and reporting bias [32,33]. Community-based studies have been employed to reduce the uncertainty in reporting a range of diseases, including water-associated diseases [34]. These methods usually involve the acquisition of data, e.g. by questionnaire possibly accompanied by biological sampling (e.g. serological surveys), in a representative population such as a retrospective cohorts or a population cross-sections. These methods can be integrated with statistical and mathematical approaches to estimate incidence. For a review of these methods, see [34] and references therein.

A common problem with these methods is that they are sensitive to the particular situation under investigation, such as country, age and social group. In addition, the climate and/or weather can have a direct impact on reporting. For example, impassable roads reducing the ability to seek medical care, and therefore detection, during the rainy season might explain the apparent seasonality of incidence of Lassa fever in humans in Sierra Leone [35]. This last example underscores the importance of integrating a variety of approaches including not only serology, lab-based sampling and statistical/mathematical models, but also participatory modelling and ethnographic research [36] to assess perceptions of risk, approaches to hygiene, health-seeking behaviour and accessibility; and how economic and social factors affect the reliability of data collection by, or reporting to, the surveillance system [35–37].

#### 3) Identifying the key risk factors/disease determinants and tackling collinearity

A key task is often to detect the main risk factors or disease determinants, and quantify their impacts. A closely related problem is collinearity (also called multi-collinearity), i.e. the situation where two or more predictor variables in a statistical model are linearly related. Collinearity might generate numerical problems, i.e. instability of parameter estimates and inflated variance of the estimated regression coefficient. In particular, collinearity often makes it impossible to attribute the effects on the response variable to the individual predictor variables [38]. This is part of the wider epistemological problem of association *vs*. causation, which is not discussed here and we refer the interested reader to, for instance, the Bradford-Hill guidelines [39].

In our context, a common source of collinearity is the highly correlated climatic variables such as temperature and rainfall. In some cases, collinearity can have a limited impact on inference, if the correlation between variables remains unchanged [40]. Patterns of collinearity between climatic variables, however, strongly depend on geographic location and environment (e.g. eco-zones) [38]; and they might vary in time due to climate change. This prevents meaningful interpretation/extrapolation of the findings beyond the geographic or environmental range of sampled data.

We share the view of Dormann et al. [38] that without a mechanistic understanding of the biophysical process, collinear variables cannot be separated by statistical means alone. This requires an understanding of the relationships between the different predictor variables, e.g. the dependence of humidity on temperature and rainfall, e.g. [41], or between the response variable and one or more predictor variables, *e*.*g*. the dependence of *Salmonella* growth on temperature.

Such mechanistic insights are not always available and one must rely on solely statistical approaches. Under this scenario, Dormann et al. [38] conducted a systematic review of methods to deal with collinearity and a simulation study evaluating their performance (in absence of mechanistic understanding) with regard to robust model fitting and prediction.

The methodologies assessed in [38], for detecting and removing collinearities include clustering (e.g. Principal Component Analysis-based Clustering, Iterative Variance Inflation Factor Analysis), cluster-independent methods (e.g. Selection of Uncorrelated Variables, Sequential Regression), latent variable regressions (e.g. Principal Component Regression, Partial Least Squares, Dimension Reduction Techniques), and a range of approaches that may be less sensitive to collinearity (e.g. Penalised Regressions, Machine-Learning methods, Collinearity-Weighted Regression). Fourier analysis is another approach that, from each time series of predictors, extracts a set of orthogonal data to be used as new descriptor uncorrelated variables [18,42]. Bayesian Network Analysis is another promising tool to identify statistical dependencies between multiple variables, and to separate these into those directly and indirectly dependent with one or more response variables [43]. This data-driven statistical tool produces a graphical network, whose structure describes the interdependency between variables [43,44]. In contrast with Path Analysis [45], Bayesian network analysis does not assume any causal relationships although this can be introduced by appropriate prior distribution for the structure of the graphical network. In particular, the method has been applied to investigate socio-economic determinants for diarrheal diseases and the role of weather in animal diseases [43,46]. Another method, applied to the 1993 Milwaukee *Cryptosporidium* outbreak [47], integrates population dynamic models with Profile Likelihood approach [48]. The problem of collinearity is removed by fixing the value of one or more parameters, and then estimating the remaining ones by maximizing the (log-) likelihood of the associated model; the approach is then repeated for a range of values of the fixed parameters. The method, which is suitable for a limited subset of the parameters, provides a better understanding of the relationship among different parameters.

#### 4) Identifying and quantifying the different sources of the temporal lag from the start of the pathway to infection to disease detection

The effects of the different meteorological, climatic, environmental and socio-economic factors on occurrence of disease are not instantaneous. Fig 1 illustrates some of the complexity. Sources of the temporal lag include the time required for potential growth of pathogen population in the environment, exposure dynamics, incubation period, and delays in reporting.

Further complications can arise from feedback from the infected population to the pathogen reservoir (*e*.*g*. rainfall facilitating contamination of fresh water from the sewage system). The required time *t*_*res*_, for the pathogen population in the reservoir to replicate and reach a sufficient value to cause infections, depends on a range of environmental and microbiological factors specific to the pathogen under investigation. Methods to estimate this time and its distribution are beyond the scope of this Review; here we simple mention some mechanistic approaches and a separate published review for temperature-driven bacterial growth in food and in water drinking systems [49–53]. The required time *t*_*exp*_, for susceptible individuals to be infected after being exposed to the pathogen reservoir, depends on the particular route of transmission and type of exposure.

The literature on microbial risk assessment framework (hazard identification, dose-response relationships, exposure assessment, quantitative risk characterization) represents an important source of methods to estimate the probability of infection and disease resulting from exposure to a variety of pathogenic microorganisms [54–56]. The effect of exposure events is, in general, distributed over a time interval. A range of approaches, based on time series analysis, has been implemented to study the distributed effects of multiple episodes of exposure on infectious outbreaks (see [57] and references therein). A general statistical framework that can simultaneously represent non-linear exposure–response dependencies (due to, for example, depletion of susceptibles) and delayed effects has been recently formulated [58].

Infections are typically revealed after the incubation period, *t*_*inc*_, (the time between infections and symptoms onsets); which is associated with patient’s physiology, whose distribution depends on the type of infection (see historical paper of Sartwell [59]). After symptoms start, only a proportion of the infected individuals seeks medical assistance (see issue above on reporting), and for only a proportion of these cases further diagnostic testing will be conducted and recorded in the public health system. This introduces a further time lag, *t*_*det*_, between the time when infected individual approaches the health system and the actual appropriate laboratory detection with diagnosis [60,61]. Even in a simple scenario, the temporal lag between the start of the pathway to infection (which can be challenging to define) and disease detection is a combination the time lags *t*_*res*_, *t*_*exp*_, *t*_*inc*_, *t*_*det*._ These are typically represented by random variables drawn from adequate distributions; for example a log-normal distribution has been proposed for *t*_*inc*_ and *t*_*det*_.

Key challenges include: these distributions are expected to be dependent on a range of factors (patient’s physiology, environment, reporting bias), they are not necessarily stationary [61], and the technical difficulties inherent with the algebra of random variables [62]. Estimating the time lag between environmental/climatic variables and infections was a common task encountered in this Review, however, none of the methods used distinguished the different sources of time lag, and in most cases the assessment was based on trial and error methods (typically, searching for high correlation between the time series of incidence and the time series of temperature and/or rainfall at 1,2, etc. weeks before the date of reported case) followed by some significance tests or selection criteria (e.g. *p*-values, AIC [63]).

Involving the wider community via community-based studies and citizen science could help in identifying the different sources of the temporal lag from the start of the pathway to infection to disease detection. This information could be used as inputs for agent based models (ABM) to simulate controlled processes in epidemics, and to assess the capability of these models to identify the multiple sources (physiology, environment, behaviour) of time lags and their statistical distributions.

#### 5) Studying the evolution of pathogen in response to climate change/variability

Very little research has investigated the potential effect of observed climate change/variability on the evolution and adaptation of pathogens [56], that is changes in the climate (e.g. mean temperature and rainfall, patterns of seasonality, etc.) on over decadal time scales. In particular, seasonality is expected to be an important driver of pathogen evolution (see [64–66] and references therein), as periods of high transmission are followed by population bottlenecks reducing strain diversity and causing rapid genetic shifts. Furthermore, external periodic perturbations (e.g. seasonality in temperature, rainfall) can resonate with the natural frequencies of the ecosystem, promoting emergency or suppression of particular strains of the pathogen [66]. Apart from the theoretical work of Koelle et al [67] which might explain the cholera strain replacement in Bangladesh due to changes in monsoon rainfall patterns, we are not aware of further research investigating evolution of water-associated pathogens in response to climate change.

#### 6) Investigating the effects of time-varying factors on transmission patterns

The importance of and challenges in understanding the effects of seasonal drivers and climate variability on the dynamics of infectious diseases are largely recognized [24,64,68–71] and not repeated here. Further challenges arise from potential changes in seasonal patterns of the drivers, for example due to control measures, and aperiodic time-varying factors [72]. Stability analysis for seasonal systems, *i*.*e*. studying the conditions for pathogen invasion and establishment in systems characterized by fluctuating environmental forcing (based for example on Floquet analysis [30,73]), represents an interesting area for future research.

#### 7) Dealing with different spatio-temporal scales

The mechanistic approaches reviewed here were in most cases deterministic compartmental models, which are strictly only valid for large epidemics. Water-associated disease outbreaks could be point-source, affecting a relatively small population. For this situation, stochastic process-based models coupled with local weather and environmental variables could be beneficial. Quantitative methodological studies applied to longer term climatic effects are limited. Extreme events, such as prolonged droughts (months or years) and heavy rain events (days or weeks), are expected to have a major impact on the dynamics of infectious diseases [74–77]. The papers reviewed here focused on the intensity of the events alone, not their frequency. Furthermore, there is no consensus on the definition of extreme weather events [77].

None of the reviewed paper investigated the long term effect of human adaptation to climatic change. The effects of Earth atmosphere range from short term weather events, to intermediate time periods events like ENSO, to longer term climate change. We are not aware of any unified approach linking together the effects of the different time-scales on water-associated infectious diseases and their spatial distribution. Spatial analysis was often performed on a temporal snapshot (cross sectional study), usually to find correlation among different variables on different locations. Only a small proportion of spatial studies included temporal dynamics, for example to study the spread of cholera in a particular region due to rainfall. Longer term changes, such as land use changes, were rarely incorporated.

## Discussion

A range of diverse methods are used to study the effect of weather and climate on water-associated diseases. Most of these methods can be connected to two main groups: (i) process *based models (PBM)*, and (ii) *time series and spatial epidemiology*. In general, PBM were employed when the bio-physical mechanism of transmission of the pathogen was known (as with *Vibrio cholerae*, the most studied pathogen). PBMs describe the progression of the infectious diseases by mimicking the bio-physical processes, typically, in terms of non-linear differential equations [14]. In contrast, statistically-oriented approaches (such as regression analysis over space or over time) tended to be used when the roles of specific environmental drivers were unclear and the methods needed to find potential correlates between environmental and/or socio-economic variables and patterns of infections.

The two clusters resemble the groups of explanatory and predictive models. Although we recognize the importance of the debate in the philosophy of science about these two groups of models [10], we avoided a formal grouping of the studies according to this classification. Applying the guidelines provided by Shmueli [10] in a rigorous and objective manner was particularly challenging in our contest. For example, many modelling studies (*e*.*g*. [78]) provide maps of the risk of a disease, i.e. they make *predictions*, by calculating the Basic Reproductive Number, which is a typical tool in *explanatory* compartmental models as built on “First Principles”. Should these models be classified as predictive or explanatory?

The use, or potential use, of the reviewed methodologies to investigate the effects of weather and/or climate is discussed in Table 3. Most of the studies focused on the short-term effects of weather as these time series of health data are more readily available.

Teasing apart the individual effects of multiple abiotic factors (e.g. weather, climate, environmental, demographic, and socio-economic) on the incidence of water-associated infectious diseases is the main challenge. Additional challenges include many other methodological problems arising from the limited understanding of the complex bio-physical mechanisms, concurrent effects of many correlated factors, poor data quality and reporting bias, and uncertainty in the knowledge of relevant parameters; these are all intrinsic limitations of the methods and the data available. Addressing these challenges would enable the formulation of a framework to understand the overall effects of weather, climate, and possibly other environmental and socio-economic factors on water-associated infectious disease.

This research has the common limitations of any systematic review. An important one is the possibility we have missed relevant studies, for example due to the failure of the search engine or studies written in languages other than English. This problem could be overcome, at least in part, by the application of snowballing procedures, i.e. recursively pursuing relevant references cited in the retrieved literature and then adding them to the search results [79]. This technique is less feasible when the initial pool of documents is as large as in the current case.

Nevertheless, considering the high number of studies included in this Review, we expect that the general patterns in our findings are robust. We recognize that there are methods developed and applied to other different classes of infectious diseases (e.g. vector-borne diseases) potentially relevant to our context which were not included in our analysis. The protocol of our review was chosen to ensure the objectivity and reproducibility of the results and the research question we identified.

### Recommendations for future research

The choice of a particular method in a study is driven by many factors, including the scientific background of the scientists involved (anecdotally, we noticed that most PBM are employed by scientists working in engineering or physics departments); the ease in implementing the methods (e.g. using freely available statistical packages); and probably the tendency to use already widely used methods, a phenomenon known as the Matthew Effect [80,81]. The choice of the methods ought to be driven by many factors, i.e. the scientific questions, the availability of data, the transmission pathways, and the bio-physical and socio-economic mechanism, as well as the state of the art of the methods. These should be critically assessed on a case-by-case basis, and not based on oversimplified, prescriptive guidelines. The findings of this Review can assist scientists in the critical selection and development of methods for quantifying the effects of weather and climate on water-associated diseases.

#### Tackling the many challenges

Important data and theoretical challenges emerged with implications for the surveillance and control of water-associated infections. The inter-connections between human health, the environment, and also animal health as advocated by the One Health holistic vision [82], are increasingly recognized. Being aware of these connections and the potential bio-physical mechanisms occurring at different spatio-temporal scales is crucial to separate out the multiple transmission pathways, to understand and quantify the different sources of the temporal lag, and to deal with collinearity. Incorporating information on human behaviour and socio-economic factors can help to reduce reporting bias, and improve understanding of the potential effect on infectious diseases of anthropogenic climate change and interventions.

#### Collecting and linking long term, high-resolution, epidemiological, socio-economic, environmental and climatic data

The integration of infection data with long-term, national-scale, environmental and land use data is an important growing approach [83]. For example, the national communicable diseases database of Public Health England has collected data from microbiology diagnostic laboratories (as well as patient’s addresses in the last five years) for England and Wales since 1989. The location of the diagnostic laboratories, or the patient residences, could be used to link cases to local weather parameters supplied by the UK Met Office in a confidential manner. These cases could also be linked with the spatial density of livestock data. The utility of these datasets could be further improved, as most current datasets are one-off surveys. Data on the spatio-temporal infection prevalence in livestock are also important information.

The paucity of this kind of information is much more pronounced for data on wildlife; with the exception of voluntary based schemes (such as citizen science [84]), we are not aware of a systematic collection of such data. Involving the wider public via community-based studies and citizen science could help in reducing the uncertainty in reporting and in identifying the different sources of the temporal lag from the start of the pathway to infection to disease detection. For example, surveys could be used by public health institutions to gather data on patient behaviour, symptom onset, food and water exposures, the likelihood of seeking medical advice, and the location of the potential sources of infection, etc.

#### Integrating bio-physical and socio-economic mechanisms of infectious disease

The emergence, risk, spread, and control of infectious diseases are affected by many complex bio-physical, environmental and socio-economic factors [1]. These include climate and environmental change, land-use variation, and changes in population and human behaviour. For example, the abundance of long term, high-resolution, surveillance data (*e*.*g*. reported infectious diseases from Public Health England) linked with local weather parameters allows the analysis of the subset of epidemiological cases when all environmental variables (except one predictor, referred to as ‘test variable’) are fixed; in this way, the problem of collinearity is naturally removed. This exercise provides a family of curves of the rates of infection, which are function of the test variable and conditioned to all other fixed predictors. From the family of curves, the potential relationship between the predictors and the rate of infection can be inferred and potentially elucidate the bio-physical mechanism.

Conversely, functional relationships between epidemiological measures (e.g. incidence) and weather parameters arising from process-based models could be used as inputs for statistical models (e.g. by providing a particular relationship for the link function in a GLM). Feedbacks from community-based studies on human behaviour could also be integrated with process-based and statistical methods to design more realistic mathematical models, which in turn can assist with making policy decisions [35].

## Supporting information

S1 Text Technical keywords/expressions used in the documents. (DOCX)

S2 Text List of search words used for the MEDLINE database. (DOC)

S1 Table List of included papers. (XLSX)

S1 Fig High resolution, zoomable image for Fig 5. (TIFF)
