# Supplementary material for: Challenges in developing methods for quantifying the effects of weather and climate on water-associated diseases: A systematic review
Source: PLoS Negl Trop Dis. 2017 Jun 12;11(6):e0005659. doi: 10.1371/journal.pntd.0005659 (PMC5481148; doi:10.1371/journal.pntd.0005659)
Supplement: S1 Text — (DOCX) [file pntd.0005659.s001.docx]

S1 Text

# Technical Keywords/Expressions used in the documents

All technical keywords and technical expression (*e.g.* generalised linear model, population dynamics) used in specific sections of each included paper were recorded in a spreadsheet cell associated with the paper.

The specific sections are: *Abstract, Keywords,* *Material and Method* (or its equivalent) in the main text and the corresponding sections in the supplementary material.

We ensured a unique semantic and orthographic form for all keywords/expression sharing the same meaning; for example all “Basic Reproductive Number“, “Basic Reproduction Number”, “Basic Reproduction Ratio”, “Basic Reproductive Ratio”, “R_0_” were recorded as “Basic Reproductive Number“. This was essential to ensure a correct analysis in Fig. 5. General keywords/expressions such as “fitting”, “differential equations”, “statistics” etc. were not recorded.

All technical keywords used in each paper are listed in the Supporting Information, S1 Table, column “Specific technical keywords and technical expression used in the paper”.

Similarly, the general methods are in the Supporting Information, S1 Table in the column titled “General methods used in the paper”.
