# Supplementary material for: Challenges in developing methods for quantifying the effects of weather and climate on water-associated diseases: A systematic review
Source: PLoS Negl Trop Dis. 2017 Jun 12;11(6):e0005659. doi: 10.1371/journal.pntd.0005659 (PMC5481148; doi:10.1371/journal.pntd.0005659)
Supplement: S2 Text — (DOC) [file pntd.0005659.s002.doc]

# S2 Text

Database: Ovid MEDLINE(R) Epub Ahead of Print, In-Process & Other Non-Indexed Citations, Ovid MEDLINE(R) Daily, Ovid MEDLINE and Versions(R)

Search Strategy:

--------------------------------------------------------------------------------

1 "water born*".ti,ab.

2 waterborn*.ti,ab.

3 "water transmi*".ti,ab.

4 "water related".ti,ab.

5 (contaminated adj3 water).ti,ab.

6 "unsafe water".ti,ab.

7 exp WATER SUPPLY/

8 "water facilit*".ti,ab.

9 (infect* adj3 water).ti,ab.

10 (unsafe adj3 water).ti,ab.

11 exp Rain/

12 rainfall.ti,ab.

13 torrential.ti,ab.

14 downpour.ti,ab.

15 aquatic.ti,ab.

16 exp Floods/

17 flood*.ti,ab.

18 exp Cyclonic Storms/

19 storm*.ti,ab.

20 tsunami*.ti,ab.

21 typhoon*.ti,ab.

22 hurricane*.ti,ab.

23 or/1-22

24 exp MATHEMATICS/

25 mathematic*.ti,ab.

26 "mathematic* model*".ti,ab.

27 "mathematic* epidemiology".ti,ab.

28 exp NUMERICAL ANALYSIS, COMPUTER-ASSISTED/

29 "numerical simulation*".ti,ab.

30 exp Computer Simulation/

31 simulation*.ti,ab.

32 exp Algorithms/

33 algorithm*.ti,ab.

34 exp Computational Biology/

35 computation*.ti,ab.

36 exp Data Interpretation, Statistical/

37 exp Factor Analysis, Statistical/

38 statistic*.ti,ab.

39 exp BASIC REPRODUCTION NUMBER/

40 "reproduction number*".ti,ab.

41 "stochastic model*".ti,ab.

42 "risk variation".ti,ab.

43 "variation in risk".ti,ab.

44 "deterministic model*".ti,ab.

45 "compartmental model*".ti,ab.

46 SEIR.ti,ab.

47 "susceptibles exposed infectious recovered".ti,ab.

48 "simple model*".ti,ab.

49 okubo.ti,ab.

50 "mechanistic model*".ti,ab.

51 or/24-49

52 or/24-50

53 23 and 51

54 23 and 52

55 54 not 53

56 limit 55 to english language
